# Viral Infections and Cutaneous Drug-Related Eruptions

**DOI:** 10.3389/fphar.2020.586407

**Published:** 2021-03-10

**Authors:** Eleonora Anci, Camille Braun, Annalisa Marinosci, Frédérique Rodieux, Elise Midun, Maria-Jose Torres, Jean-Christoph Caubet

**Affiliations:** ^1^ Pediatric Allergy Unit, University Hospitals of Geneva and University of Geneva, Geneva, Switzerland; ^2^ Pediatric Allergy Unit, University Lyon 1 Claude Bernard, Villeurbanne, France; ^3^ Division of Clinical Pharmacology and Toxicology, University Hospitals of Geneva, Geneva, Switzerland; ^4^ Allergy Unit, Hospital Regional Universitario de Málaga, Universidad de Málaga, Ibima-Bionand-Aradyal, Málaga, Spain

**Keywords:** drug, hypersensitivity, allergy, virus, mechanism

## Abstract

In the general population, up to 10% of children treated by antibiotics have cutaneous adverse drug reaction, but allergy is confirmed in less than 20% of patients. Most of the non-allergic reactions are probably due to virus, such as enterovirus acute infection or Ebstein-Barr Virus (EBV) acute infection or reactivation. Especially in children, viruses have the propensity to induce skin lesions (maculopapular rash, urticaria) due to their skin infiltration or immunologic response. In drug-related skin eruptions, a virus can participate by activating an immune predisposition. The culprit antibiotic is then the trigger for reacting. Even in severe drug-induced reactions, such as Drug Reaction with Eosinophilia and Systemic Symptoms (DRESS) syndrome, viruses take part in immune phenomena, especially herpes viruses. Understanding the mechanisms of both virus- and drug-induced skin reaction is important to develop our clinical reflection and give an adaptive care to the patient. Our aim is to review current knowledge on the different aspects and potential roles of viruses in the different type of drug hypersensitivity reactions (DHR). Although major advances have been made those past year, further studies are needed for a better understanding of the link between viruses and DHR, to improve management of those patients.

## Introduction

Drug allergy is a major public health problem, associated with a high morbidity and mortality, as well as elevated medical costs (Macy, 1998; MacLaughlin et al., 2000; Solensky, 2013; Solensky, 2014; van Dijk et al., 2016). The clinical pictures, and the underlying mechanisms are very heterogeneous (Macy, 1998; MacLaughlin et al., 2000; Solensky, 2013; Solensky, 2014; van Dijk et al., 2016). Thus, diagnosis of drug allergies is difficult and a challenge for the treating physician (Macy, 1998; MacLaughlin et al., 2000; Solensky, 2013; Solensky, 2014; van Dijk et al., 2016). A further problem is overdiagnosis. It is common, particularly during childhood, as the drug allergy may be transient and allergy tests are difficult, cumbersome, of limited sensitivity and expensive. One of these confounding factors are virus infections, as they constitute the major cause of skin eruptions in childhood and represent an important differential diagnosis in patients with a suspicion of drug allergy (Goodyear et al., 1991). Indeed, common clinical manifestations of drug allergy i.e., maculopapular exanthema and urticaria, are similar to viral-induced rashes. Some viral infections are name-giving for drug-induced exanthemas (rubeola like or measles like exanthemas) and distinction is difficult during the acute phase. Avoidance of the potential incriminated drug is usually recommended, although “threating through” can be considered as an option with close monitoring of the patient.

In addition, viral infections may be involved by providing a co-factor for immune stimulation. Numerous clinical observations suggest that viral infections promote or aggravate drug-related skin rashes (Ponvert et al., 1999; Shiohara and Kano, 2007; Caubet et al., 2011). Epstein Barr Virus (EBV) is one of the best known examples with a higher rate of skin eruptions in EBV-infected patients treated by betalactams (BL) antibiotics (Chovel-Sella et al., 2013). Another example is the apparent role of herpes viruses in the pathogenesis of severe drug-related reactions, particularly in the Drug Reaction with Eosinophilia and Systemic Symptoms (DRESS), which is increasingly discussed in the literature (Descamps et al., 2001; Kano et al., 2006; Shiohara et al., 2006).

Based on a selection of best quality papers, the aim of this manuscript is to review current knowledge on the different aspects and potential roles of viruses in the different types of drug hypersensitivity reactions (DHR).

## Pathomechanisms

### DHR Classification

The traditional classification of Rawlings and Thompson proposed a sub-classification of adverse drug reactions (ADR) into type A reactions, which are due to the pharmacological activity of the drug (80% of all ADR). Type B reactions comprise about 15–20% of all ADR: they involve DHR (Rawlins, 1981).

The DHR have been shown to be induced by different and distinct mechanisms. The drug or drug metabolite usually acts as a hapten, which is able to bind by covalent bonds to a protein and thus forms an antigen that is able to induce IgE- or T cell-mediated allergic reactions (White et al., 2015). Drugs can also stimulate the immune system directly, namely by binding by non-covalent bonds (pharmacological interaction) to immune receptors like HLA or T-cell receptor (TCR); this so-called p-i mechanism stimulate exclusively T-cells (Pichler et al., 2002).

The third mechanism is summarized as “pseudo-allergy,” term that is controversial, where the drug interferes with inflammatory mechanisms or activates inflammatory cells like mast cells, eosinophils, neutrophils, etc. without involving the specific immune system. Such pseudo-allergic reactions manifest as clinical pictures mimicking allergy, depending on the cells/mediators involved: e.g., the mast cells with urticarial/anaphylaxis are involved in off-target pharmacological activities of certain drugs on mast cells receptors (MRGPRX2); the blocking of enzymes like cyclooxygenase in nonsteroidal anti-inflammatory drugs (NSAID) can lead to exacerbated asthma or urticaria; and blocking the degradation of bradykinin by angiotensin converting enzyme (ACE) inhibitors may lead to angioedema.

### Mechanisms of Viral-Induced Skin Eruptions

Skin eruptions are among the most common causes of consultations at primary care physicians, particularly paediatricians: it has been found that up to 17% of paediatric emergency consultations are motivated by occurrence of a skin eruption (Kramkimel et al., 2010; Landolt et al., 2013). The major causes are infections, most notably viruses. Despite the relatively high frequency of this problem, epidemiologic data are scarce (Folster-Holst and Kreth, 2009a). The estimated prevalence of maculopapular virus-linked exanthemas is estimated to be 158.3/10,000 (CI: 142.3–174.4) (Vega Alonso et al., 2003). Based on typical morphological feature, six classical exanthemas have been described at the beginning of the 20th century, i.e., measles or rubeola, scarlet fever, rubella, Filatow–Dukes disease (fourth disease), erythema infectiosum (fifth disease), and exanthem subitum (sixth disease) (Keighley et al., 2015). Exanthemas not included in the previous list are referred to “atypical exanthemas” (Drago et al., 2012). The majority of exanthema are caused by non-polio enteroviruses, respiratory viruses (adenoviruses, rhinoviruses, parainfluenza viruses, respiratory syncytial virus, influenza viruses), acute EBV, human herpes viruses (HHV) 6 and 7, parvovirus B-19 and norovirus (Hogan, 1996; Leiste et al., 2008). Among enterovirus, the most commonly involved are Coxsackie virus A16 and EV71, responsible for hand, foot and mouth disease, typically in children (He et al., 2017). Different clinical aspects have been described based on the morphological aspects of primary lesions (i.e., erythematous, papular, vesicular, urticarial-like, pustular, or petechial) and the most common types are maculopapular exanthema and maculovesicular exanthema (Schneider et al., 2013).

The mechanisms by which a virus leads to the development of skin eruption have been explored since the 60s (Mims, 1964; Mims, 1966). They are complex and are still not well defined in many aspects. The occurrence of a rash induced by a virus may depend on virus ability to grow in dermal and epidermal cells. Indeed, viruses are able to infiltrate skin and infect tissue cells, via fixation to cellular receptors or intracellular penetration (Laksono et al., 2016). Particularly, it has been shown that skin manifestations can be induced in part by a direct viral cytopathic effect (inclusions, ballooning, vacuolation and necrosis) which may lead to macroscopical modification such as edema and hemorrhage, generating the skin lesions (Geck et al., 1964; Agol, 2012). Theoretically, any circulating virus, free or cell-associated, which localizes in a skin blood vessel can infect the vessel wall (or pass through) and grow in extravascular tissues, giving rise to a skin eruption (Mims, 1966). Skin cell lesions induce discharge of pro-inflammatory products, especially damage (or danger) signals, cytokines and chemokines (Smith, 1972; Folster-Holst and Kreth, 2009b). Keratinocytes are probably important actors of non-specific inflammation, through the fixation of the virus and the secretion of different signals (Strittmatter et al., 2016). In addition to the direct effect of the virus, immunologic mechanisms induced by the virus can also be involved in the development of a skin lesion. Indeed, viral-induced cell-mediated responses might be responsible for damage through a nonspecific inflammatory reaction (Parham and Janeway, 2009). Recruitment of adaptive immune cells is permitted by the interaction between inflamed endothelium receptors and skin-addressing markers on the lymphocyte surface, for example the CLA (Cutaneous Lymphocyte Antigen) (Schon et al., 2003; Clark, 2010).

From another point of view, viruses can also lead to exanthema by a local delayed (type 4) hypersensitivity reaction within the dermis to various pathogens, such as in Gianotti-Crosti syndrome, where exanthema is typically papulo-vesicular, but neither viral particles nor antigens have been demonstrated in the skin lesions (Gianotti, 1979). This syndrome would results from an immunologic response rather than a primary manifestation of an infection (Lowe et al., 1989; Magyarlaki et al., 1991; Hofmann et al., 1997; Folster-Holst and Kreth, 2009b).

However, it is unknown why skin rashes are seen in only a small proportion of all generalized virus diseases, and the characteristic distribution of skin lesions in different virus exanthema remains unclear (Mims, 1966). Genetic and individual susceptibility may play an important role to the development of skin lesions and should be taken into account to understand the complexity of the problem. Non-immune mechanisms (i.e., sensitivity to histamine, antigen-antibody complexes clearing by reticuloendothelial system) may be involved as personal immunological factors necessary to develop an allergic reaction (Levine, 1965).

### Potential Interaction Between Virus and Drug

**FIGURE 1:**
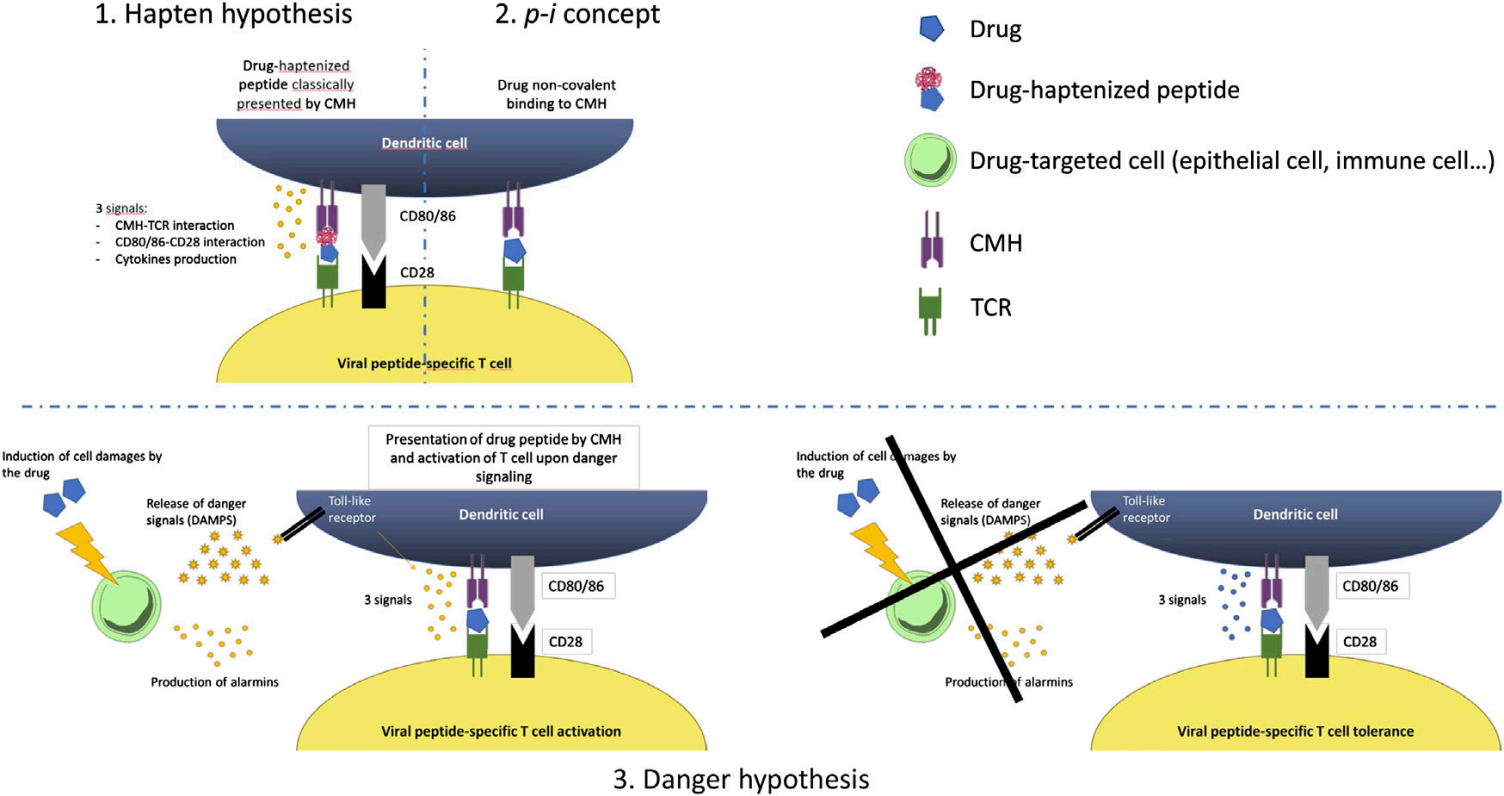
Potential immune mechanisms involved in the interactions between viruses and drug.

The interaction between virus immunity and drug hypersensitivity are multiple and complex (White et al., 2015) (Figure 1). The heterologous immunity models is an enlarged vision that takes into account the specific HLA-restriction and the minimal co-stimulatory signals observed in drug-related Severe Cutaneous Adverse Reactions (SCARs) (White et al., 2015). In this model, drug is supposed to induce the formation of a neo-antigen recognized by virus-specific memory T cells. Those T cells were earlier sensitized by life-long infecting viruses, which periodically sort out of latency and turn on transcriptional programs (White et al., 2015). This intermittent viral replication stimulates a substantial anti-viral specific T cell proliferation, without developing the functional unresponsiveness which normally follows recurrent infections (Virgin et al., 2009). In this model, memory T cell are generated following pathogen exposure and reside at specific anatomic sites. These memory T cells may cross react with haptenated endogenous peptides presented in the context of the HLA risk allele, or drugs that bind the TCR and/or MHC in a non-covalent manner following the p-i model, or an altered repertoire of endogenous peptides following drug binding to MHC (Todd, 2006).

Another theory that explain this interplay between drug and infection is the danger hypothesis which was firstly proposed by Matzinger since the early 1990s (Das et al., 2011). This model states that the primary driving force of the immune system is to protect against danger (Anderson and Matzinger, 2000). Presentation of an antigen in the absence of danger results in tolerance, while the presence of a danger signal will result in a full-blown immune response. Indeed, three different elements are needed to elicit an immune response. Signal 1 represents the interaction between the MHC-restricted antigen and the T-cell receptor. Signal 2 is represented by the co-stimulatory molecule–receptor interactions and a series of pro-inflammatory cytokines such as IL-2, TNF-α, and IFN-γ that act indirectly on antigen presenting cells to up-regulate the expression of co-stimulatory molecules. Signal 3 represents polarizing cytokines that act directly on T-cells, and lead to either TH1 or TH2 immune responses. The danger signal can result from chemical, physical or viral stress. This theory was proposed to partially explain the reactions in HIV patients.

Regarding IgE-mediated hypersensitivity reactions, there is no data in the literature indicating a link between viruses and IgE mediated drug reactions. However, the implication of viruses in IgE-mediated food allergy is well-known and similarly, a potential role of viruses in these reactions is probable (Muraro et al., 2014). Further studies are needed to explore this important aspect.

## Role of Virus in Benign Nonimmediate Reaction

### Viral Infection as a Differential Diagnosis

A common situation in clinical practice, and particularly in pediatric, is the appearance of a benign exanthema or urticaria (i.e., without any danger signs) in patients treated by antibiotics, mainly BL, and NSAID (Bigby, 2001; Thong and Tan, 2011).

It is difficult to distinguish urticaria-like exanthemas from “classical” urticaria, which is characterized by wheal and flare reactions: in “classical” urticaria, the manifestation is acute after drug intake (min to hours) vs. urticaria-like exanthemas, which appear after days, often together with macular exanthematic lesions. Classical urticarial lesions last <24 h, while some form of the urticaria-like exanthemas (some linked to drug intake) persists longer (e.g., as maculopapular exanthems). In clinical practice, most of these patients are labeled as drug allergic without appropriate testing, mainly due to fear of a life-threatening reaction, leading to an overdiagnosis of drug allergy. However, it has been found that an allergy will be confirmed by a complete allergy workup in only 7–20% of those patients (Caubet et al., 2011; Ponvert et al., 2011; Rubio et al., 2012; Demoly et al., 2014).

The cause of those non-allergic eruptions in patients with a negative allergy workup has been poorly investigated until recently, particularly in the paediatric population. By including 88 children who had developed an exanthema during a BL treatment, Caubet et al. detected a virus by PCR or serology in 65.9% of the children with a negative drug provocation test (DPT), the most frequent being enteroviruses (Picornavirus) (Caubet et al., 2011). Similarly, Atanaskovic-Markovic et al. found that 333 children (22%) tested positive for a virus or *Mycoplasma pneumoniae* infection among 1,026 children with a suspicion of nonimmediate hypersensitivity reactions (Atanaskovic-Markovic et al., 2016). Only two of them were confirmed to be allergic to the culprit drug (Atanaskovic-Markovic et al., 2016). This suggests that in patients developing an exanthema or delayed-appearing urticaria while taking concomitantly a drug, viral infection is frequent; and that reaction to the drug taken can be detected only rarely. Possibly the combination of viral infection—facilitating the drug reaction, is transient, and the single drug may be tolerated. The virus infections would represent the co-stimulatory factor enhancing drug reactions.

However, in these studies, a virus has not been found in all patients with a skin eruption during a BL treatment. It can be explained by the fact that not all viruses have been tested in those studies. From another point of view, we cannot exclude that the positivity of PCR or serology was due to a previous infection or an acute infection without any link with the current rash.

Clinically it is very difficult, and often impossible to differentiate a rash of viral origin or secondary to a drug allergy. Although blood tests are not routinely performed in our current clinical practice for exanthema or urticaria, it has been recently suggested that some tests could be helpful to distinguish between viral- and drug-induced skin eruptions. As an example, Hari Y et al. have shown that in viral exanthemas, IFN-γ is increased in most serum samples from different acute viral diseases, while in drug-induced exanthemas, IL-5 alone or in combination with granzyme B and perforin are often found to be increased – together with some eosinophilia (Hari et al., 1999; Bellini et al., 2013). Another example is the potential role of thymus and activation-regulated chemokine (TARC/CCL17) which plays an important in TH2 immune responses. Thus, a link between serum TARC levels and HHV-6 reactivation in patient with DRESS has been found and serum TARC levels have been suggested to be a useful indicator to differentiate DRESS/DIHS with HHV-6 reactivation from other drug eruptions (Ogawa et al., 2014).

### The EBV Example as a Co-Factor for Drug-Induced Skin Eruptions

The best illustration for the drug-related exanthemas during a viral infection is those occurring after antibiotic administration in patients with an acute EBV infection. Indeed, it has been shown that the incidence of skin rash is higher in EBV patients treated by antibiotic (typically ampicillin) compared to EBV patients without associated antibiotic treatment (i.e., 27.8–90% and 3–10%, respectively) (Pullen et al., 1967; Copeman and Scrivener, 1977; Luzuriaga and Sullivan, 2010). No association with age, gender, ethnicity or allergic history appears to be correlated with rash development after antibiotic treatment in EBV patients (Chovel-Sella et al., 2013).

One of the hypothesis regarding the mechanisms for the development of skin eruption occurring in patients with infectious mononucleosis and concomitantly treated by antibiotics, appears to be a transient virus-mediated immune alteration (Thompson and Ramos, 2017). In patients with EBV infection, the CD8^+^ T cell population is typically expanded, leading to the secretion of INF-γ and interleukine-2 (IL-2). This has been shown to inhibit the TH2-response (IL-4, 5, 6, 9, 13) (Schissel et al., 2000; Banerjee et al., 2014) and the anti-inflammatory IL-10 secretion, while the TH1-response is activated (Onodi-Nagy et al., 2015). These alterations could set the stage for a loss of antigenic tolerance and the development of a reversible DHR (Shiohara and Kano, 2007). Thus, the administration of an antibiotic, especially ampicillin, would then be the trigger for activation of this anti-IL-10 pro-TH1 response, leading to the maculopapular rash (Thompson and Ramos, 2017).

Conversely, recent studies suggest that a true long lasting antibiotic hypersensitivity might be a lot more prevalent than previously thought, during the acute EBV infection in patients treated by amoxicillin (Renn et al., 2002; Onodi-Nagy et al., 2015). Some authors found positive lymphocyte transformation tests (LLTs) to the incriminated antibiotic (Renn et al., 2002), as well as positive delayed intradermal and patch-tests in those patients (Jappe, 2007; Onodi-Nagy et al., 2015). Authors also described positive DPT or severe DHR upon re-exposure to the beta-lactam at distance of the initial reaction (Jappe, 2007). Thus, it is recommended to assess these reactions with a complete allergic workup, and discuss a DPT.

Long lasting HS may be supported by EBV which continuously co-activates immune response and prevents apoptosis of drug specific T-cell, as it has been found in EBV-induced malignant diseases (Chen, 2011). This anti-apoptotic capacity of EBV could be responsible to the maintenance of lymphocytes, which will then be activated by antibiotic administration (Chen, 2011; Lindsey et al., 2016).

Interestingly, it has been suggested that ampicillin can directly induce the reactivation of EBV, leading to a skin eruption. Thus, Saito-Katsuragi et al. reported the case of a 23-year-old woman with a Still’s disease, who developed a maculopapular rash after an ampicillin treatment. She developed serum IgG antibody against EBV-VCA 1 week after. The authors performed two DPT with intravenous ampicillin, resulting in a recurrence of the maculopapular rash 24–48 h after the treatment intake. They monitored the concentration of EBV DNA in blood and found a significant increase of EBV DNA levels after the injection of ampicillin and just before the appearance of the skin rash. Further studies are needed to confirm the hypothesis by which ampicillin would be responsible for a reactivation of EBV, which would then trigger the skin eruption.

EBV continues to be one of the most important models to understand interaction between drugs and concomitant acute or chronic viral infections. Lymphocyte stimulation and direct stimulation of the virus appears to be the most likely hypotheses. However, further researches are needed for a better understanding of the mechanisms involved in the dysregulation of the immune system, leading to a reaction.

### ROLE OF VIRUS IN SEVERE NONIMMEDIATE REACTIONS

A variety of severe, rare, potentially life-threatening, drug reactions are described, for which recent evidences suggest an intimate relationship with reactivation of specific virus: the DRESS syndrome, the Stevens-Johnson syndrome (SJS) as well as the Toxic epidermal necrolysis (TEN) and transitional forms (Tohyama and Hashimoto, 2011).

### DRESS Syndrome

The DRESS syndrome is a drug-induced delayed reaction with an estimated incidence ranging from one case among 1,000 to 10,000 drug exposures (Fiszenson-Albala et al., 2003). It is most frequently associated with administration of aromatic anticonvulsants, antidepressants, sulfonamides and sulfones, anti-inflammatory drugs, antibiotics, angiotensin-converting enzyme inhibitors and beta-blockers (Kardaun et al., 2013). It has been suggested that viruses play an important role in the physiopathology of DRESS (Redwood and et al., 2018). Hypotheses are based on the evidence of virus replication (primo-infection or reactivation) during the development of disease (Descamps et al., 2001; Ichiche et al., 2003; Picard et al., 2010). Human herpes virus 6 (HHV-6) was the first chronic persistent virus incriminated in the pathology of DRESS (Descamps et al., 1997), being now considered, for some, as a specific and sensitive diagnostic criteria (Shiohara et al., 2007; Watanabe, 2018).

However, the role of HHV replication remains controversial as a study did not find a significant correlation between HHV DNA load and DRESS diagnosis (Ushigome et al., 2012). Several studies reported that HHV replication does not occur early in the clinical course of DRESS and generally, viremia is observed greater than 2 weeks following symptoms onset (White et al., 2015). These data suggest that viral reactivation itself is not involved in the onset of DRESS, but rather than some viruses, in particular of the herpes group, may be involved in the prolonged clinical course of DRESS (Ishida et al., 2014).

The expansion of CD4^+^ T cells and CD8^+^ T cells during HHV-6 reactivation seems to be an important feature in many patients with DRESS’s multiple organ failure (Pritchett et al., 2012). In addition, it has been found that patients with HHV-6 reactivation have significant higher serum levels of TNF-α, compared to patients without HHV-6 reactivation. *In vitro* and *in vivo* studies showed that TNF-α and other cytokines participate in reactivation of CMV through the induction of CMV immediate early gene expression, leading to the initiation of the viral replication. CMV IE gene has a high level of homology with HHV-6 U95 gene and it is possible that TNF-α interacts identically with it (Watanabe, 2018). The serum thymus and activation-regulated chemokine (TARC) levels are also found to be higher in DRESS patients with HHV-6 replication than those without. TARC may be able to directly activate HHV-6 through a TARC receptor, or induce a relative immunosuppression through the activation of regulatory T cells (Tregs) (Watanabe, 2018). This is in accordance with some observations of dysfunction of Tregs and plasmacytoid dendritic cells in the DRESS syndrome (Takahashi et al., 2009). Thus, there are some evidence that HHV-6-related mechanisms exist to explain at least partially the complications of DRESS.

The importance of drug exposure could be integrated with those of viral interplay in a recent model: the heterologous immunity model. Furthermore, active viral replication is not required in this abovementioned model, so the evidences of viral reactivation highlighted during SCARs development may just represent a tangential event. There is still a need of further studies to highlight differences between patients with or without viral reactivation. In this context, a retrospective case series of 29 pediatric patient with DRESS, reported that those who were HHV-6 positive experimented a significantly greater severity and a longer hospitalization compared to HHV-6 negative subjects (11.5 days vs. 5 days, *p* = 0.039) (Ahluwalia et al., 2015). Even in adults, patients with HHV-6 reactivation showed longer course and more severe organ involvement than others, suggesting a possibly prognostic significance of HHV-6 (Tohyama et al., 2007; Asano et al., 2009).

Further researches should also emphasize on reactivation of other latent viruses too. Apparently, viral activation follows an identifiable chronological pathway and seems to implicate several viruses in the present order: firstly EBV and/or HHV-6, followed by HHV-7 and soon after CMV (Cho et al., 2017). The simultaneous appearance of multiple concomitant viral reactivations would be explained by the ability of herpes virus to reactivate others virus. The role of the EBV in the development of multi-organ involvement of DRESS is discussed particularly because infectious mononucleosis-like symptoms are observed during the early phase of DRESS (Tohyama and Hashimoto, 2011). Furthermore, Mardivirin et al. investigate the possibility of a drug-induced flare-up of DRESS due to antibiotic prescription. Amoxicillin seemed to be an aggravating factor, probably due to the same pathomechanism of amoxicillin-induced rash in EBV infected patients (Mardivirin et al., 2010).

Finally, hypothesis for DRESS syndrome pathophysiology include interaction between different factors: 1) genetic susceptibility factors, such as HLA type or cytochrome p450 polymorphism (Cho et al., 2017); 2) viral infection (primo-infection or replication) inducing a particular pre-activated immune state; and 3) drug as a final trigger for the immune reaction. Virus reactivation could also be the trigger for relapse of DRESS syndrome (Tan and Chan, 2016), as seen in chronic diseases. Besides, it is interesting to note that similarities are highlighted between DRESS and autoimmune disease mechanisms (Michels and Ostrov, 2015).

### SJS and TEN

Similar observations have been made in SJS and TEN. These syndromes are most commonly caused by DHR rather than viruses (such as EBV, CMV, HHV-6, HSV, Varicella zoster virus, hepatitis A virus and HIV) (Stutman, 1987; Werblowsky-Constantini et al., 1989; Lam et al., 2004; Bay et al., 2005; Pereira et al., 2007; Cruz et al., 2010; Wetter and Camilleri, 2010; Khalaf et al., 2011; Kunimi et al., 2011; Kim et al., 2012; Sotelo-Cruz, 2012; Ferrandiz-Pulido and Garcia-Patos, 2013; Irungu et al., 2017). In about 30% of cases of SJS and TEN, no causative drug is identified, and in 15%, drug responsibility is deemed unlikely (Duong et al., 2017). Since now, over 200 drugs have been associated with SJS/TEN, most commonly sulfonamides and BL antibiotics (Roujeau et al., 1995; Forman et al., 2002; Sheridan et al., 2002).

To date it is still not clear if the virus is a potential co-factor or trigger. Expression of viral DNA fragments in the keratinocyte layer could lead to activation of CD4^+^ T-helper cells, which induce various reactions, including cytokines production and subsequent inflammatory responses (McDermott et al., 2013). Furthermore, infections activate systemic host inflammatory pathways, as consequence, a perturbation of the natural defense mechanisms of oxidase enzymes could occur and multisystem damages may follow (Bay et al., 2005). Despite everything, F. Brunet-Possenti reports a case of SJS during a primary EBV infection in a 17-year-old adolescent. A 10 years retrospective study presented by Forman confirmed it, founding as the most commonly incriminated infectious agent the herpes simplex virus (19.7%) (Forman et al., 2002). However, while HHV-6 reactivation is primary related to DRESS, it is rare in SJS/TEN (Neuman et al., 2013), sometimes observed in patients treated with anticonvulsant (Peppercorn et al., 2010; Teraki et al., 2010). Actually, researchers are still arguing if “drug-induced” SJS/TEN and “infection-related” SJS/TEN are two separate entities.

### HIV Example

Human immunodeficiency virus (HIV) infection is a long-life latent virus hosted by CD4 T cells and macrophages (Zack et al., 1990). This viral infection is associated with important immune deregulations and higher rates of conditions requiring drug administration. It has been found that frequency of DHR in HIV-infected patients is particularly high, up to 100 times more common compared to HIV-negative subjects (Coopman et al., 1993; Rzany et al., 1993; Temesgen and Beri, 2004). The pathogenesis and the reason for the greater propensity for HIV-infected patients to develop DHR to a great variety of drugs that can be particularly severe, remain unknown. It may be related to their greater exposition to medication compared to general population and/or to a higher incidence of co-infection with EBV and CMV (Cytomegalovirus) (Smith et al., 1997; Todd, 2006; Hoosen and et al., 2019). Since many different drugs are involved, the viral infection appears to enhance drug reactivity in general, not only for specific drugs.

This infection itself leads to apparent decrease and loss function of T cells in the blood and skin, in addition to dysregulation of tolerance to self-antigens (Todd, 2006). Interestingly, the incidence of severe DHR in the HIV-infected population has also been reported to increase with increasing stage of the disease, i.e., decreasing CD4^+^ T cells counts and CD4/CD8 ratio (Coopman et al., 1993; Arp et al., 2005). An interesting example is the hypersensitivity reaction to Trimethoprim-Sulfamethoxazole (TMP-SMX), which occurs in 40–80% of HIV infected individuals (Meyer et al., 2015). The patients with uncontrolled HIV replication have a decrease reduction capacity and a depletion of glutathion in the CD4 cells, leading to an increased toxicity of nitrososulfamethoxazole (n-SMX), a reactive and toxic metabolites of SMX (Correia et al., 2002). This modification in redox balance may be related to the Tat protein, an HIV-specific protein essential for the viral replication (Das et al., 2011). The Tat protein would be secreted by infected cells, in relation to the viral load and disease progression, and promotes drug reactions, increasing oxidation status (Meyer et al., 2015). This strong predisposition to drug reactions is clearly dependent to multiple factors linked to the immune deregulation associated to the primary infection (Todd, 2006). But our understanding of the exact pathomechanisms remains limited and requires further studies.

The higher frequency of allergic drugs reactions in this viral infection may be the result of increased levels of cytokines and cell-surface markers and thereby acting in concert with the drug antigen, amplifying the potential of a drug to cause an immune reaction (Pirmohamed et al., 2002). Although an attractive hypothesis when applied to the pathogenesis of DHRs, there are many questions that remain unanswered. Indeed, the lack of direct experimental evidence has led to heavy criticism of the danger hypothesis (Jozefowski, 2016).

## Role of Virus in Other Type of DHR

### The NSAID Example

It has recently been reported that NSAID could be the most common cause of DHR in children (Woessner et al., 2002; Morales et al., 2015). Prevalence of self-reported hypersensitivity to NSAID has been shown to range from 0.6 to 5.7% in the general population (Dona et al., 2011). NSAIDs, including aspirin, are a group of drugs sharing the capability of inhibiting the cyclooxygenase (COX) enzymes responsible for the prostaglandin synthetase pathway of arachidonic acid metabolism. The pathogenesis of hypersensitivity reactions owing to cross‐intolerance has been hypothesized to be related to COX‐1 inhibition, although it has not been clearly demonstrated (Macy, 1998).

Interestingly, it has been suggested that blocking prostaglandin synthesis could also allow specific cytotoxic lymphocytes to produce asthma attacks during respiratory tract viral infections (Szczeklik, 1988). Correlation between viral illness and NSAIDs hypersensitivity was first theorized by Szczeklik (1988). As cytotoxic lymphocyte activity is normally inhibited by prostaglandin E2 (PGE2); in case of aspirin and other NSAIDs treatment, COX enzyme is blocked and PGE2 production decrease allowing cytotoxic lymphocytes to attack and eliminate the respiratory tract cells infected by the virus. As a result, lysosomal enzymes and mediators are released and this could precipitate a NSAIDs reaction. These acute attacks can be prevented by avoidance of all drugs with anti-cyclooxygenase activity. However, asthma continues to run a protracted course because of chronic viral infection (Szczeklik, 1988). Nakagawa et al. suspected an acquired analgesic idiosyncrasy secondary to viral infection. They observed anti-Herpes simplex virus (HSV) IgG antibodies titers and hypothesized a relationship between the serological evidence of HSV infection and positive bronchial hyperresponsiveness provocation tests (Nakagawa et al., 2001). Contrariwise, several studies have showed that NSAID can inhibit viral replication (Newton, 1979; Pereira et al., 2003; Reynolds and Enquist, 2006; Zimmermann and Curtis, 2017), yielding more difficult the interpretation of virus and NSAID interaction.

## Conclusion

In addition to be a major differential diagnosis of DHR, viruses might interact in different ways in different types of DHR to unmask a latent drug allergy. Particularly, viruses have been shown to cause cellular damages, to increase the inflammatory response, to induce the production of specific antibodies, to provoke a change in antigenic expression and to stimulate T-cell replication. From another point of view, the drug might enhance viral replication, leading secondarily to skin eruption. Pathomechanism of viral-induced skin lesions has been poorly studied. However, a better understanding is of major importance, as it can provide major insight in the understanding of drug induced skin rashes. Further studies are urgently needed to clarify the role of viruses in drugs HSRs, to improve the management of patients presenting skin eruptions during treatments and to avoid useless drug avoidance, related with increased morbidity and mortality.

## Abbreviations:

ADR, adverse drug reaction; APC, antigen presenting cells; BL, betalactam; COX, cyclooxygenase; CYP, cytochrome P; DRESS, drug rash with eosinophilia and systemic symptoms; HIV, human immunodeficiency virus; HSV, herpes simplex virus; DIHS, drug induced hypersensitivity syndrome; NSAID, nonsteroidal anti-inflammatory drugs; PGE2, prostaglandin E2; SJS, Stevens-Johnson syndrome; SMX, sulfamethoxazole; n-SMX, nitrososulfamethoxazole; TMP, trimethoprim; TEN, toxic epidermal necrolysis; EBV, Epstein-Barr virus; EV, enteroviruses; RSV, respiratory sincitial virus; GCS, Gianotti-Crosti syndrome; MI, mononucleosis infectious; NRTI, nucleoside reverse transcriptase inhibitor; HR, homing receptor; CLA, cutaneous lymphocyte-associated antigen; SAg, superantigen; PRR, pattern recognition receptor; SCAR, severe cutaneous adverse reactions syndrome; DPT, drug provocation test.
